# Assessing the Influence of Temperature Changes on the Geometric Stability of Smartphone- and Raspberry Pi Cameras

**DOI:** 10.3390/s20030643

**Published:** 2020-01-23

**Authors:** Melanie Elias, Anette Eltner, Frank Liebold, Hans-Gerd Maas

**Affiliations:** Institute of Photogrammetry & Remote Sensing, TU Dresden, 01069 Dresden, Germany; anette.eltner@tu-dresden.de (A.E.); frank.liebold@tu-dresden.de (F.L.); hans-gerd.maas@tu-dresden.de (H.-G.M.)

**Keywords:** MEMS, smartphone camera, Raspberry Pi camera, camera calibration, photogrammetry, interior orientation, low-cost camera

## Abstract

Knowledge about the interior and exterior camera orientation parameters is required to establish the relationship between 2D image content and 3D object data. Camera calibration is used to determine the interior orientation parameters, which are valid as long as the camera remains stable. However, information about the temporal stability of low-cost cameras due to the physical impact of temperature changes, such as those in smartphones, is still missing. This study investigates on the one hand the influence of heat dissipating smartphone components at the geometric integrity of implemented cameras and on the other hand the impact of ambient temperature changes at the geometry of uncoupled low-cost cameras considering a Raspberry Pi camera module that is exposed to controlled thermal radiation changes. If these impacts are neglected, transferring image measurements into object space will lead to wrong measurements due to high correlations between temperature and camera’s geometric stability. Monte-Carlo simulation is used to simulate temperature-related variations of the interior orientation parameters to assess the extent of potential errors in the 3D data ranging from a few millimetres up to five centimetres on a target in X- and Y-direction. The target is positioned at a distance of 10 m to the camera and the Z-axis is aligned with camera’s depth direction.

## 1. Introduction

Smartphones have become indispensable in modern human life as they are not just purely communication tools. They are qualified for citizen science applying photogrammetry due to built-in cameras enabling the acquisition and processing of geolocated image data directly on the device. The global increase of climate-related natural hazards [1] demands new technologies to support their observation, detection and forecasting to improve early-warning systems. The progress in smartphone technology creates new possibilities in this regard. Current devices comprise high storage capacity, large processing power, a wide range of built-in sensors and high-resolution cameras. Therefore, they are already a centrepiece in several early warning systems that are supported by volunteered geographic information with user-generated content [2,3]. Recently published water-level monitoring and flood-forecasting tools adapt well-established photogrammetric methods to smartphone- and Raspberry Pi (RPi) cameras to use them as versatile measurement instruments, e.g., [4,5,6,7,8]. To restore the collinearity between the 2D image and the related 3D object scene, i.e., to determine the linear relationships of 2D image points and 3D object points that lie on image rays with a shared origin called projection centre, knowledge about the interior orientation parameters (IOP) is required, which can be determined via photogrammetric camera calibration, e.g., [9,10,11].

A calibration is valid as long as the camera geometry does not change. Alternating IOP can be caused by aperture- or focus adjustment on the one hand or due to physical impacts such as strong motion on the other hand, e.g., [12,13]. In addition, [14,15,16,17] have shown that ambient temperature changes greatly influence the IOP of webcams, digital single-lens reflex (DSLR) cameras and bridge cameras resulting in image shifts and zooming effects. However, there is still a knowledge gap regarding the relationship between changing temperatures and the interior geometry of low-cost cameras based on the micro-electro-mechanical system (MEMS) technology built in smartphones or used as RPi cameras. Information is missing about error quantities that must be expected when these cameras are considered for measuring purposes. The cameras use smallest sensors (diagonals much smaller than 1 cm) resulting in small pixels with a size of about 1 µm. They are equipped with simple, focusable lenses with fixed focal lengths of a few millimetres. The sensors are glued to the sensor plate to achieve small device sizes. As stated by [18], “the performance of a MEMS device can be strongly affected by thermal stresses resulting from constraining interactions among device’s multiple layers and between the package and the device“. Referring to the camera design, changes of the camera temperature may have strong impact on the camera geometry stability and thus on the measurement accuracies compared to e.g., DSLR cameras. With regard to water-level monitoring applications described by [4,5], deviations in the IOP will cause errors in the translation of 2D water lines measured in images to 3D object space. These errors range from a few millimetres to several centimetres depending on the camera-to-object distance. Apart from measurement applications provided for environmental monitoring, temperature-induced variations of the camera geometry are considerable issues in, e.g., machine vision [14,15], automotive [19] or medicine [20].

This study provides a comprehensive investigation to examine the impact of self-heating and ambient temperature changes with regards to the interior camera geometry to further assess possible measurement errors. Self-heating impacts are expected for smartphones where the camera is firmly integrated close to components emitting heat such as the battery, the display or the central processing unit (CPU). Uncoupled low-cost cameras, e.g., RPi cameras, are assumed to be less affected by self-heating effects. Due to their potential for outdoor monitoring applications [21], they might be exposed to ambient temperature changes that can range from strong heat due to direct solar radiation to strong cold due to icing or snow.

In previous work, Refs [20,22] made extensive investigations on camera warming effects on image acquisition. The authors provide different approaches to correct image drifts resulting from self-heating and ambient temperature changes. In this respect, they consider two types of cameras. Firstly, cameras with interchangeable lenses, where the projection center is independent from the sensor and thermal expansion affects only the sensor plane. Secondly, cameras with directly mounted lens and sensor board, e.g., mobile phone cameras, where thermal expansion affects both, the image plane and the projection center. In the latter case, Refs [20,23] suggest that image drifts are only related to changes of the camera position and orientation, i.e., the exterior orientation parameters (EOP). However, in this study it is assumed that especially the IOP of low-cost cameras are prone to temperature variation due to the camera design. Thus, this study explains on the example of two smartphone cameras and one RPi camera, how to use single-image camera calibration based on spatial resection to observe the IOP while the investigated camera is exposed to temperature changes. Furthermore, Monte-Carlo simulations are used, considering the changed IOP due to temperature variations, to estimate the effect of errors on image measurements transferred into object space.

## 2. Hardware

The primary built-in cameras of the smartphones LG Google Nexus 5 and Samsung Galaxy S8 were used to investigate temperature changes on the IOP stability due to self-heating, and the original RPi camera module v2.1 with a fixed focal length of 3 mm was used to evaluate the impact of ambient temperature fluctuations (detailed device specifications are given in Table 1).

The reason why the cameras of two smartphones were investigated are their device characteristics affecting the temperature inside the smartphone housing and thus the camera temperature. First, Nexus devices use plain Android operation systems in contrast to other manufactures who implement own user interfaces or background services resulting in higher processor load and higher heat emission. Secondly, Samsung’s Galaxy S8 is used representative for smartphones with built-in cooling systems. It is highly likely that the heatpipe-cooling system has significant influence on the camera temperature. In short, thermal energy is absorbed from sources emitting heat, e.g., the CPU, and transferred to lower temperature ends. In this case, these temperature ends are close to the camera sensor. Furthermore, the cases of both devices are completely different, which influences heat dissipation (Nexus 5: plastic back and frame [27]; Galaxy S8: glass back, aluminium frame [28]). In summary, it is assumed that the cameras of both devices will react differently to self-heating-induced temperature changes.

### 2.1. Smartphone Camera Application

In the following experiments, an in-house smartphone camera application was used, which is based on the framework Open Camera 1.3.8 [29], providing full control over the camera. Options were implemented to disable autofocus and to fix the focus at a defined distance using the Android camera API 2 [30]. The application further enables the activation of several sensors to increase the workload, which results in self-heating of the smartphone. During image acquisition the battery and the CPU temperature are recorded. Background tasks that are unrelated to the measurements are cancelled.

## 3. Methods and Algorithms

### 3.1. Single-Image Camera Calibration

A method was developed to monitor the IOP continuously while the investigated device is exposed to temperature changes to study camera stabilities during heating and cooling. Photogrammetric camera calibration allows to determine the camera parameters using a single image of a 3D test field with a large number of targets with known reference coordinates. Taking serial images of the 3D test field permits nearly continuous determination of the camera parameters during ambient temperature changes.

#### 3.1.1. IOP Estimation

**Figure 1:**
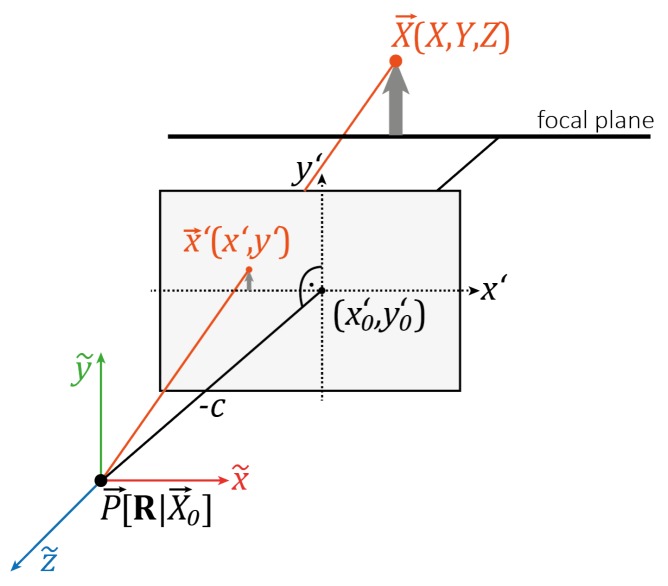
Transformation of a 3D object point $\vec{X}$ into a 2D image point $\vec{x{}^{\prime }}$ where $\vec{P}\left[R|\vec{X_{0}}\right]$ is the camera projection center in a world coordinate system.

The photogrammetric calibration strategies imply the determination of the IOP, thereby solving a non-linear equation system of collinearity equations, which describe the transformation of a 3D object point into a 2D image point (see Figure 1).

This transformation is described with:

(1) $$\left(\begin{array}{c} \tilde{x}\\ \tilde{y}\\ \tilde{z} \end{array}\right)=R^{T}\left(\vec{X}-\vec{X_{0}}\right)$$

where $\vec{X}={\left(X,Y,Z\right)}^{T}$ is a 3D object point in the world reference system that is transformed into the camera coordinate system $\left(\tilde{x},\tilde{y},\tilde{z}\right)$ utilizing the EOP given by a 3 × 3 rotation matrix $R^{T}\left(r_{i,j}\in R^{T}\right)$ and a 3D translation vector $\vec{X_{0}}={\left(X_{0},Y_{0},Z_{0}\right)}^{T}$ to the camera projection centre in object space. The 2D image coordinates can be derived in the camera coordinate system with:

(2) $$x{}^{\prime \prime }=\frac{\tilde{x}}{\tilde{z}};y{}^{\prime \prime }=\frac{\tilde{y}}{\tilde{z}}$$

(3) $$\begin{array}{c} x{}^{\prime }=x_{0}{}^{\prime }-c\cdot x{}^{\prime \prime } \end{array}$$

(4) $$\begin{array}{c} y{}^{\prime }=y_{0}{}^{\prime }-c\cdot y{}^{\prime \prime } \end{array}$$

where $\vec{x{}^{\prime }}={\left(x{}^{\prime },y{}^{\prime }\right)}^{T}$ are coordinates of the 2D image point and $c,x_{0}{}^{\prime },y_{0}{}^{\prime }$ are IOP with the principal distance *c*, where $-c=z{}^{\prime }$, and the principal point $x_{0}{}^{\prime },y_{0}{}^{\prime }$. Usually, camera lenses are influenced by lens distortion that has to be considered in the point-to-point transformation. Lens correction terms $\Delta {x{}^{\prime }}_{rad},\Delta {y{}^{\prime }}_{rad}$ (radial lens distortion) and $\Delta {x{}^{\prime }}_{dec},\Delta {y{}^{\prime }}_{dec}$ (decentering lens distortion) are added to the 2D image coordinates, which are adapted from Browns standard camera model [31]:

(5) $$x{}^{\prime }=x_{0}{}^{\prime }-c\cdot \left(x{}^{\prime \prime }+\Delta x{}^{\prime }\right)$$

(6) $$y{}^{\prime }=y_{0}{}^{\prime }-c\cdot \left(y{}^{\prime \prime }+\Delta y{}^{\prime }\right)$$

with:

(7) $$\Delta x{}^{\prime }=\Delta {x{}^{\prime }}_{rad}+\Delta {x{}^{\prime }}_{dec}$$

(8) $$\Delta y{}^{\prime }=\Delta {y{}^{\prime }}_{rad}+\Delta {y{}^{\prime }}_{dec}$$

and:

(9) $$r^{2}=x{{}^{\prime \prime }}^{2}+y{{}^{\prime \prime }}^{2}$$

(10) $$\Delta {x{}^{\prime }}_{rad}=x{}^{\prime \prime }\cdot \left(a_{1}\cdot r^{2}+a_{2}\cdot r^{4}+a_{3}\cdot r^{6}\right)$$

(11) $$\Delta {y{}^{\prime }}_{rad}=y{}^{\prime \prime }\cdot \left(a_{1}\cdot r^{2}+a_{2}\cdot r^{4}+a_{3}\cdot r^{6}\right)$$

(12) $$\Delta {x{}^{\prime }}_{dec}=b_{1}\cdot \left(r^{2}+2\cdot x{{}^{\prime \prime }}^{2}\right)+2\cdot b_{2}\cdot x{}^{\prime \prime }\cdot y{}^{\prime \prime }$$

(13) $$\Delta {y{}^{\prime }}_{dec}=b_{2}\cdot \left(r^{2}+2\cdot y{{}^{\prime \prime }}^{2}\right)+2\cdot b_{1}\cdot x{}^{\prime \prime }\cdot y{}^{\prime \prime }$$

The resulting equations are commonly known as collinearity equations [32] extended by lens correction terms [33]:

(14) $$x{}^{\prime }=x_{0}{}^{\prime }-c\cdot (\frac{r_{11}\cdot \left(X-X_{0}\right)+r_{21}\cdot \left(Y-Y_{0}\right)+r_{31}\cdot \left(Z-Z_{0}\right)}{r_{13}\cdot \left(X-X_{0}\right)+r_{23}\cdot \left(Y-Y_{0}\right)+r_{33}\cdot \left(Z-Z_{0}\right)}+\Delta x{}^{\prime })$$

(15) $$y{}^{\prime }=y_{0}{}^{\prime }-c\cdot (\frac{r_{12}\cdot \left(X-X_{0}\right)+r_{22}\cdot \left(Y-Y_{0}\right)+r_{32}\cdot \left(Z-Z_{0}\right)}{r_{13}\cdot \left(X-X_{0}\right)+r_{23}\cdot \left(Y-Y_{0}\right)+r_{33}\cdot \left(Z-Z_{0}\right)}+\Delta y{}^{\prime })$$

The unknown camera parameters are derived solving a overdetermined non-linear collinearity equation system considering least-squares adjustment based on spatial resection. The solution of this equation system requires initial estimates of the camera parameters as well as of image observations of known 3D object points (also known as reference points). The determination of six EOP parameters $\left(X_{0},Y_{0},Z_{0},\omega ,\phi ,\kappa \right)$, where ω,ϕ,κ are 3-axis Euler rotation angles that can be expressed by rotation matrix $R^{T}$, and eight IOP $\left(c,x_{0}{}^{\prime },y_{0}{}^{\prime },a_{1},a_{2},a_{3},b_{1},b_{2}\right)$ requires at least seven 3D reference points that provide 14 observations, i.e., the measured image coordinates $\left(x{}^{\prime },y{}^{\prime }\right)$. To avoid singularities, the reference points have to be spatially distributed, i.e., they cannot lie in one plane. Furthermore, the reference points and the projection centre must not be located on a danger surface, e.g., a cylinder [33]. Spatial point distribution is also necessary if the lens distortion has to be described because this requires format-filling image observations. The quality of spatial resection is assessed calculating the standard deviation of the unit weight ${\hat{s}}_{0}$, which represents the accuracy of the image measurements. Moreover, the individual standard deviation of each investigated parameter ${\hat{s}}_{k}$ as well as information about the correlation between the parameters are derived from the corresponding variance-covariance matrix (e.g., [34]).

Single-image camera calibration permits the continuous investigation of camera parameters but impedes the direct differentiation into camera-internal and camera-external variations due to correlations between the IOP and EOP. Thus, changes in the exterior geometry (related to housing deformations) will be reflected in the IOP as well as changes in the interior geometry (related to sensor-to-lens deformations) will be reflected in the EOP. As can be taken from the literature, correlations are mainly found between the depth direction and the principal distance $t_{z}\Leftrightarrow c$ as well as the principal point and the EOP $x_{0}^{\prime },y_{0}^{\prime }\Leftrightarrow t_{x},t_{y},t_{z},\omega ,\phi ,\kappa$ [33,35]. Exemplary for this, [14] point out in their study on temperature-related image shifts that the principle point and the translation parameters $t_{x},t_{y}$ show similar motion patterns, which is mainly related to correlations between the involved parameters. For that reasons, one should be cautious with conclusions about the origin of camera effects in interpreting changes of the camera model. In the following experiments, the EOP were fixed to avoid superimposing relative changes of the continuously estimated IOP, e.g., due to residual errors of the EOP or correlations between IOP and EOP.

#### 3.1.2. Designing the 3D Test Field

The 3D test field consists of 60 spatially distributed, partially coded markers, including four “out-of-the-plane” reference points in different depths (see Figure 1, right). The test field itself was calibrated via camera self-calibration prior to the experiments following the calibration scheme given by [35]. The image data required for this was captured with the DSLR camera Nikon D700 (28 mm fixed focal length) and processed with the photogrammetry software AICON 3D studio v12.0 resulting in 3D reference point coordinates, which were determined with mean standard deviations of 7.8, 7.9 and 15.2 µm in x-,y- and z-direction, respectively. The image measurement accuracy, given by ${\hat{s}}_{0}$, amounts to 7.6 µm.

#### 3.1.3. Data Acquisition and Processing

At the beginning of each experiment, the investigated camera was fixed in a stable position with a temperature invariant mount. For that purpose, a carbon tripod was combined with a smartphone camera mount that fixed the device from all sides according to the device frame. A gauge stand with insulated holders and clamps was used to fix the RPi camera in front of the test field. Once the camera was mounted, it was manually focussed looking straight at the test field with a format-filling image configuration. The focus was not changed by the operator during one measurement series. Spatial resection was used to determine both, the IOP and the EOP using approximations for the IOP whose determination is trivial (principal distance c≈ nominal focal length, principal point $x_{0}{}^{\prime },y_{0}{}^{\prime }\approx 0$, radial distortion $a_{1},a_{2},a_{3}\approx 0$, decentring distortion $b_{1},b_{2}\approx 0$ [33]). Parameters for the EOP were determined within the calibration of the test field. The behaviour of the camera parameters was now observed using images of the 3D test field for spatial resection, which were taken in a sequence with an interval of ten seconds while the observed camera was exposed to temperature changes. In this way, the IOP as well as the stochastic models were determined according to the number of images while the EOP were fixed after the first measurement. The required reference points were measured within the calibration images using a subpixel accuracy image point measurement tool implemented in AICON 3D Studio v12.0.

**Figure 2:**
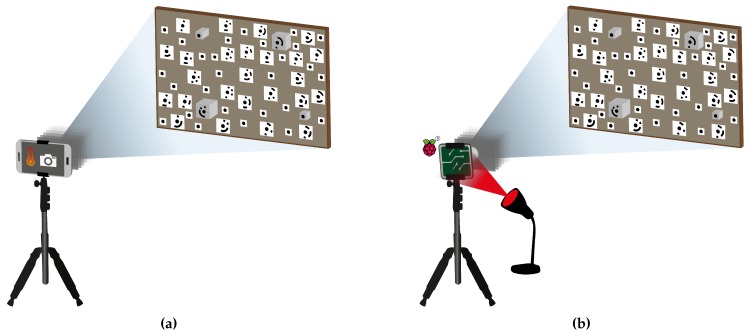
Measurement setup to investigate the relation between the IOP and temperature variations occurring from (**a**) self-heating and (**b**) ambient temperature variations using smartphone cameras and low-cost RPi cameras, respectively. Camera-to-object distance: about 90 cm. Test field dimensions: 70 × 50 cm, Marker diameter: 10 mm.

Smartphone self-heating was caused by the implemented camera application as described in Section 2.1 whereas ambient temperature changes were provoked externally by alternating the radiation intensity of a thermal infrared lamp (which was turned on and off for approximately ten minutes) pointed at the RPi camera. To quantify changing temperatures, the smartphone CPU temperature was logged each time an image was captured, and a temperature sensor DHT 11 was installed at the back of the RPi camera, which was connected to a RPi computer that triggered the camera and requested the temperature each time an image was shot. The experimental setup is visualised in Figure 2 for both heating sources.

The relation between the IOP of each camera and the temperature change was investigated with at least two consecutive measurement series M1 and M2 in different scenarios for each device keeping the same camera geometry. For the smartphone cameras eight measurements were made in total, two series each for cold- and warm start-ups of the two devices. During the cold start-up image acquisition starts immediately after switching on the device, and during the warm start-up data capture starts shortly after a warm-up period. These two different approaches have a strong impact on the initial device temperature and thus the temperature amplitude during device heating. Each smartphone camera took 150 images during self-heating of the device. The RPi camera took 250 images, while it was alternately heated and cooled using the infrared lamp. Thereby, the lamp was left turned on until temperature was not changing anymore (at a temperature of about 60 ${}^{\circ }$C). Afterwards, the lamp was turned off until temperature did not change again (25–30 ${}^{\circ }$C). The IOP were determined for each image of the entire sequence via spatial resection.

### 3.2. Simulating the Impact of Differently Changing IOP at Measurements in 3D Object Space

If changes of the camera geometry occur due to temperature changes, it is important to estimate the impact at measurements in 3D object space [36,37,38]. The Monte-Carlo simulation was used to evaluate how different changes of the camera geometry affect errors in object space. Especially, Monte-Carlo simulation allows to consider the complex interaction between the individual IOP and temperature in relation to the accuracy of image-based measurements transferred into 3D object space. Therefore, sets of k(k∈N) parameters, reflecting the IOP, are randomly generated *n*-times (n∈N) considering residuals and mathematically correlations, to project *n* regular grids of image points onto a virtual object plane in 3D object space. As indicated by [36,38], IOP-related variations in object space are highly correlated with the reference object that is used for intersection and “should be as close as possible to the expected object products of the photogrammetric application of interest” [36]. If this is not considered, more degrees of freedom related to the object scene are introduced that might mitigate or intensify IOP-related variations due to depth variations of the reference object. In this study, the focus is not at one specific application. Therefore, the usage of a plane is the most general way to provide information about the point scattering even if the depth component cannot be considered. Details about the implementation are provided in Appendix A allowing for an application-specific adaptation of the simulation.

In this case, 50.000 multivariate random vectors $\vec{X_{k}}\left(c,x_{0}{}^{\prime },y_{0}{}^{\prime },a_{1},a_{2},a_{3},b_{1},b_{2}\right)$ are considered when a raster of nine image points is projected onto a virtual object plane at a depth, i.e., camera-to-object distance, of *Z* = 10 m. The image rays intersect the object plane in a defined distance. Thereby, iteratively changing camera geometries cause shifts in the virtual object plane leading to scattered intersection points. The error magnitudes of the projected points in object space are defined by the principal standard deviations in *X*- and *Y*-direction as well as the maximum and mean distances of the scattered object points to the projected object points of a camera with error-free IOP (see Appendix A).

## 4. Results and Discussion

The subsequent section investigates how changing temperature affects the IOP separated in self-heating and ambient temperature impacts. The obtained knowledge is used to simulate temperature-related changes of the camera parameters in order to assess the error metric in object space.

### 4.1. Self-Heating Temperature Impacts at Smartphone Cameras

**Figure 3:**
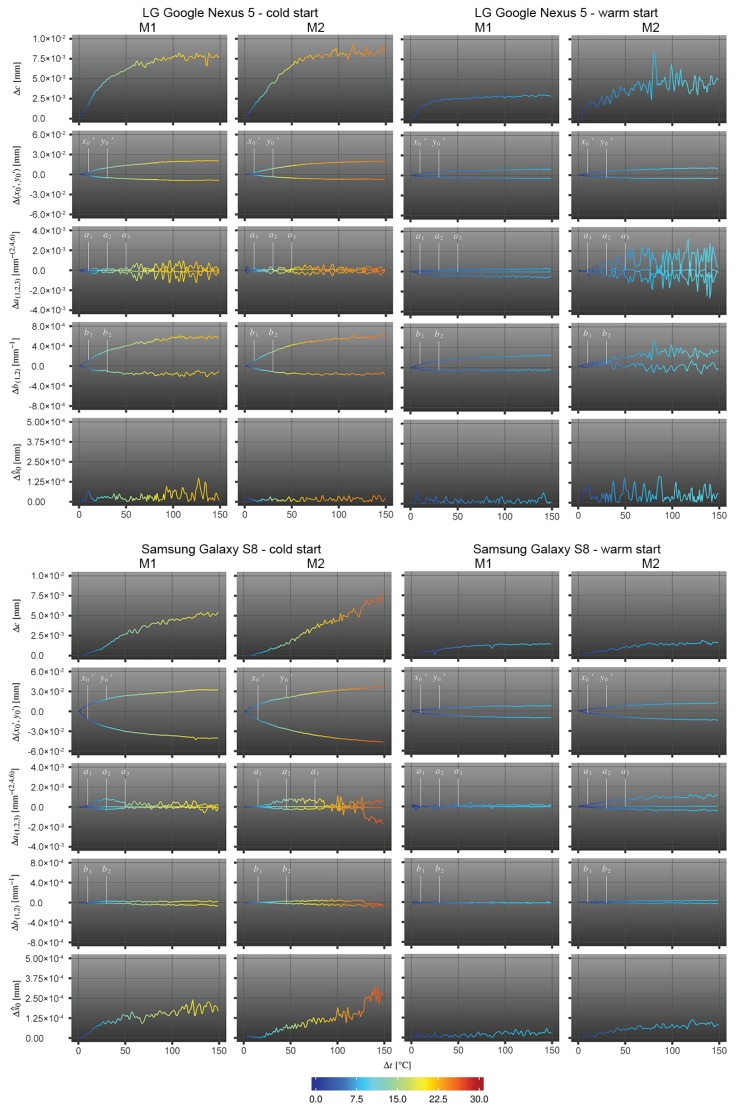
IOP-to-temperature assessment; x-axis: number of measurements per measurement series M1/M2; y-axis: estimated deviation per parameter Δk compared to the respective initial value; color-code: temperature difference Δt (measured at the battery) to the initial temperature value when the phone was started.

Cold-started and warm-started cameras are considered as two individual cameras to be investigated. Table 2 shows the deviations between the last and the first estimated variables after 150 measurements (25 min of heating), respectively for each investigated camera and two measurement periods M1, M2. In Figure 3, the difference of the estimated IOP to the expected parameters, corresponding to the initial values when the cameras were not affected by temperature variations, are visualised. In addition to this, the differences of the standard deviation of the unit weight $\Delta {\hat{s}}_{0}$ are visualised that indicate possible changes of the measurement accuracy.

**Figure 4:**
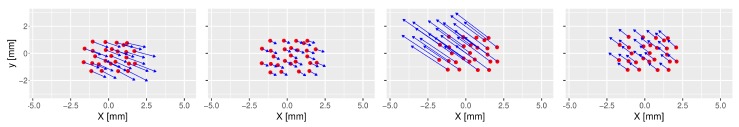
Visualisation of image point shifts and zooming effects between the first (red dots) and the last (heads of the blue arrows) measurement within measurement series M1 respectively for (from left to right) the LG Google Nexus 5 camera (cold-started, warm-started) and for the Samsung Galaxy S8 camera (cold-started, warm started). The arrow length is superimposed by a factor of 50.

All smartphone experiments reveal that the higher the rise in temperature, the more the principal point $\left(x_{0}{}^{\prime },y_{0}{}^{\prime }\right)$ is shifting and the more the principal distance *c* is increasing. These effects are also visible in Table 3, where the image content seems to move although the camera device, i.e., the smartphone, was fixed. Figure 4 confirms the changes in the principal distance and the principal point resulting in directional zooming effects. Similar observations were made by [14]. Focussing on the different camera types, the principle point of the LG Google Nexus 5 camera moves to the lower right, whereas the principle point of the Samsung Galaxy S8 camera moves to the upper left. This may be related to the mounting direction of the built-in camera sensors that may be rotated by 180${}^{\circ }$. Having cold started cameras with a strong increase of the device temperature, the changes in the IOP are significantly higher compared to warm started cameras. The principle point of the Samsung Galaxy S8 camera is changing nearly twice as much as the principle point of the LG Google Nexus 5 camera (about 40 vs. 16 pixels in terms of cold started cameras and 11 vs. 8 pixels in terms of warm started cameras). It is highly likely that this is related to the greater temperature increases of the Samsung device compared to the Google Nexus smartphone that was already expected from the different hardware designs. This finding would support the assumption of housing deformations affecting the exterior orientation of the camera module and/or internal camera deformations due to different kinds of heat dissipation.

The extent to which the principal distance *c* is changing is influenced by the magnitude of temperature change and shows similar results for both tested cameras (average deviation is about 0.007 mm at cold start and 0.003 mm at warm start). This would mean a depth of field variation of 5 mm and 3 mm (Nexus/ S8) for cold started- and 3 mm and 2 mm (Nexus/S8) for warm started devices assuming a camera whose focus distance was set to 1 m. These changes in the captured images lead to decreasing image point measurement quality, which becomes obvious by the increasing noise reflected in the standard deviations ${\hat{s}}_{0}$ and in the measurements of especially the principal distance and the radial lens distortion in the later measurements. It can be observed that the IOP changes towards an equilibrium, which was also observed by e.g., [14,20,39], when smartphones are protected against overheating by reducing the CPU load. Moreover, the camera parameters and the temperature reveal a linear relationship that is further investigated in Section 4.3.

Considering the increase of measurement uncertainties, it is important to evaluate if the estimated IOP-variations are significant. For that purpose, temperature-related two-sided moving variances $s_{k}^{2}$ are calculated over $n_{1}$ consecutive measurements for each investigated camera parameter *k*. They are compared to the two-sided moving averages of $n_{2}$ squared standard deviations ${\hat{s}}_{k}^{2}$ of each investigated camera parameter *k* (see Section 3.1) via f-test to examine if $s_{k}^{2}$ is significantly greater than ${\hat{s}}_{k}^{2}$. Usually, f-test requires measurements with a normal distribution, but a large sample size (n>30) can excuse violations of the normality assumption according to [40]. The size of the moving window was set to $n_{1,2}=51$. The test parameters are given in Table 4 assuming a significance level of α=0.05.

The f-test was performed for each time stamp of one measurement series (provided that the moving variance could be calculated over $n_{1}=n_{2}$ measurements) summarising the number of success. Success means that the null hypothesis could be rejected, i.e., the temperature-related variances are significantly greater than the measurement uncertainties and thus significant. The success ratios ζ(k) (number of success divided by the total number of tests) are given in Table 5 summarising the test results from measurement series M1 and M2, respectively.

The results indicate that variations due to temperature changes are significant with regards to individual measurement series. In a few individual measurements, where $H_{0}$ could not be rejected, measurement uncertainties are greater than temperature-related deviations. This is usually the case when the test field drifted out of the focus resulting in an insufficient estimation of the image coordinates and thus leading to higher measurement uncertainties.

### 4.2. Temperature Impacts at the Stability of RPi Cameras

**Figure 5:**
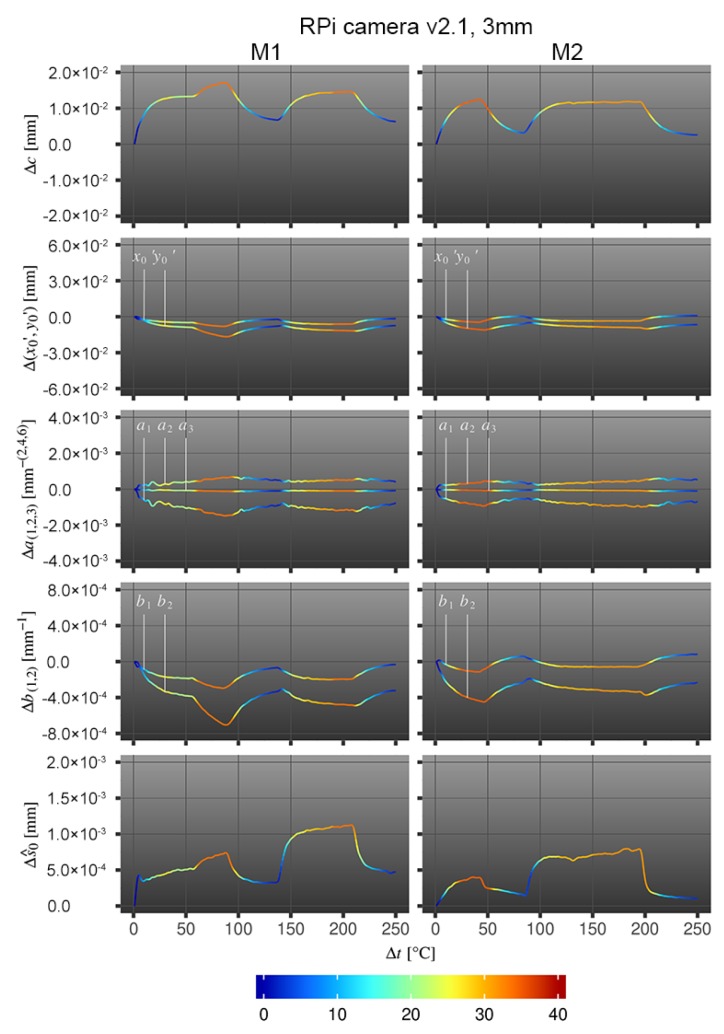
IOP-to-temperature assessment with RPi camera v2.1, which was exposed to alternating temperature, evaluating two measurement series (M1, M2); x-axis: number of measurements per measurement series; y-axis: estimated deviation per investigated parameter compared to the initial value; color-code: temperature difference Δt to the initial temperature value before the red-light radiation lamp was switched on for the first time.

Influences of changing temperatures at the camera stability of RPi cameras are shown in Figure 5.

The relation between the individual IOP changes due to camera exposure to heating and cooling are compared to the initial values using the same approach as for the smartphone camera Section 4.1. The estimated changes in the IOP of RPi camera v2.1 with 3 mm lens are highly correlated with temperature changes in both measurements, which is further examined in Section 4.3.

**Figure 6:**
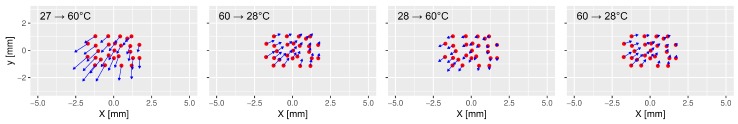
Visualisation of image point shifts and zooming effects considering the turning points between the heating and cooling phases of the RPi camera investigation M1. The arrow length is superimposed by a factor of 50.

For the RPi camera module, back and forth focus shifts due to expansion and contraction of the principal distances *c* because of alternating temperatures are revealed. The principal point ($x_{0}{}^{\prime },y_{0}{}^{\prime }$) is changing as well. When the temperature rises, the point moves into one direction (lower left) and when the temperature decreases, the point moves almost completely back along the same direction. Both can be seen in Table 6 and Figure 6; the image content moves wave-like and is out of focus when temperature rises and again in focus when temperature decreases. It is of special interest that the image points do not return to their starting position when the temperature changes to its initial state. For that purpose, some permanent changes of the camera geometry due to temperature changes must be assumed either due to changes of the relationship between sensor board and projection center or due to camera movements. Similar to the smartphone cameras, the changing interior geometry causes strong fluctuations in the image point measurement accuracies, which results in lower reliabilities of the estimated parameters when the camera is exposed to direct radiation. The influence of the temperature changes at the measurement accuracy can be seen towards the standard deviation of the unit weight ${\hat{s}}_{0}$ which is up to 3.5 times higher at the maximum temperature compared to the initial measurement accuracy. These conclusions are also confirmed by f-test, which was performed in the same way as for the smartphone measurements (see Section 4.1). The success rates ζ(k) amount to ζ(c)=0.96, $\zeta (x_{0}{}^{\prime })=0.96$, $\zeta (y_{0}{}^{\prime })=0.89$, $\zeta (a_{1})=1.00$, $\zeta (a_{2})=1.00$, $\zeta (a_{3})=1.00$, $\zeta (b_{1})=0.75$ and $\zeta (b_{2})=0.80$.

### 4.3. Statistical Evaluation of Temperature Dependencies

**Figure 7:**
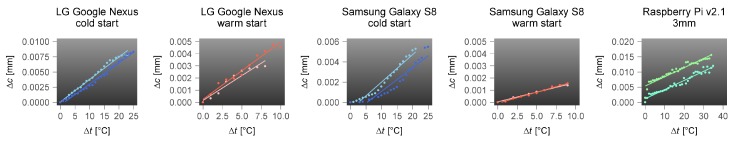
Visualisation of the linear dependencies between changing IOP and temperature on the example of principal distance *c*. Considering the RPi observations, the aggregated data is sorted by temperature change. Light colors refer to the respective measurement series M1 and darker colors refer to the respective measurement series M2.

The experiments reveal a linear relationship between temperature changes and the determined IOP (see Figure 7). To assess the statistical relevance of the relation between temperature change and IOP stability, the Pearson correlation coefficients ρ are calculated for the estimations of the the interior orientation parameter *k* and the simultaneously measured temperature *t*. To estimate the significance of the correlation coefficient between independently estimated variables, t-test is applied to determine the significance levels given by the *p*-values (must be less than α=0.05).

**Figure 8:**
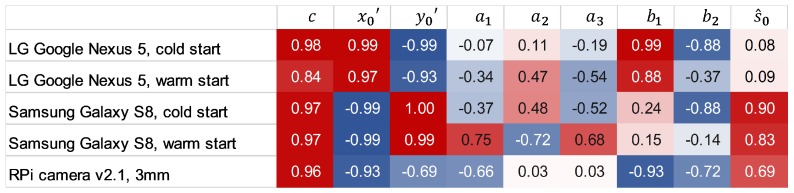
Correlation between changing IOP and changing temperature given by Pearson’s correlation coefficient ρ, calculated from the measurement series M1 and M2 for each camera.

A high correlation of nearly 100% between temperature and principal distance *c* as well as principal point ($x_{0}{}^{\prime },y_{0}{}^{\prime }$) is revealed in this study (see correlation matrix in Figure 8). Thereby, reversed correlations of the principal point coordinates $x_{0}{}^{\prime }$ and $y_{0}{}^{\prime }$ (except for the RPi camera) close to ρ±1.0 are noticeable. Moderate correlations between temperature and radial lens distortion parameters $a_{1},a_{2},a_{3}$ are observable. Furthermore, strong correlations between temperature and decentering lens distortion, described by $b_{1},b_{2}$, are noticeable for the RPi camera and the smartphone camera integrated in the LG Google Nexus 5. It is worth mentioning that the measurement accuracies ${\hat{s}}_{0}$ of the Samsung Galaxy S8- and the RPi measurements are highly correlated with the temperature but not the measurements made with the LG Google Nexus 5 camera. The reason can be found considering the image clips given in Table 3 and the parameter deviations shown in Figure 3. The images of LG Google Nexus 5 appear to be less effected by focus changes than the images of Samsung Galaxy S8. One reason might be that the direction of movement of the principal point of the camera of the LG Google Nexus 5 counteracts the extension of the principal distance whereas the moving direction of Samsung Galaxy S8’s principal point amplifies the impact of the change of the focus (see Figure 4). Together with the correlation coefficients, *p*-values were determined which were less than the significance level α=0.05 in all calculations. Thus, the determined correlations are considered to be significant for all assessed parameters.

**Figure 9:**
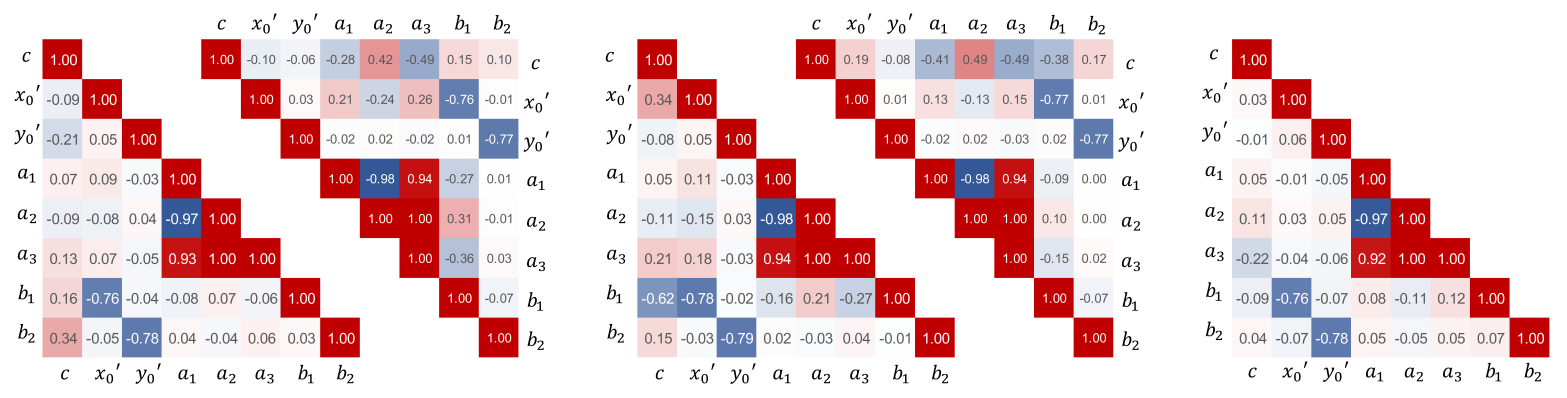
Median correlations $\tilde{\rho }$ between the individual IOP determined for each camera using the observations of measurement series M1 and M2. From left to right, LG Google Nexus 5 cold-started, warm-started; Samsung Galaxy S8 cold-started, warm-started, Raspberry Pi camera v2.1.

Using Monte-Carlo simulations to assess temperature-related measurement errors in object space requires knowledge about the correlations between the IOP, although they are reduced as far as possible by using an adapted 3D test-field- and camera configuration. The correlations were obtained from the variance-covariance matrices, which were also calculated during camera parameter determination. The correlations between the parameters should be consistent within the measurements of one measurement series because of a constant camera configuration. However, temperature-related measurement uncertainties resulted in noise of the correlation coefficients. The noise amounts to ρ±0.01 using warm started smartphone cameras and the RPi camera. With regards to cold started smartphone cameras, the noise is getting bigger at the end of the measurement series when the temperature increase is at its highest. To further obtain one significant value to use in the subsequent Monte-Carlo simulations, the median values were determined considering all observations in both given series M1 and M2 (see Figure 9).

Most parameters of the IOP are less- or completely uncorrelated. Significant correlations are reported between the parameters of the radial lens distortion ($a_{1}\Leftrightarrow a_{2}\Leftrightarrow a_{3}$) and between the principal point and the parameters of the decentering lens distortion ($x_{0}^{\prime },y_{0}^{\prime }\Leftrightarrow b_{1},b_{2}$). As described by [33], these mathematically correlations are related to the principle of camera calibration and cannot be avoided. However, all estimated correlations are considered in the Monte-Carlo simulations to ensure plausible sets of IOP in agreement with temperature-induced changes.

### 4.4. Temperature-Related Error Assessment in Object Space: Results of Monte-Carlo Simulation

**Figure 10:**
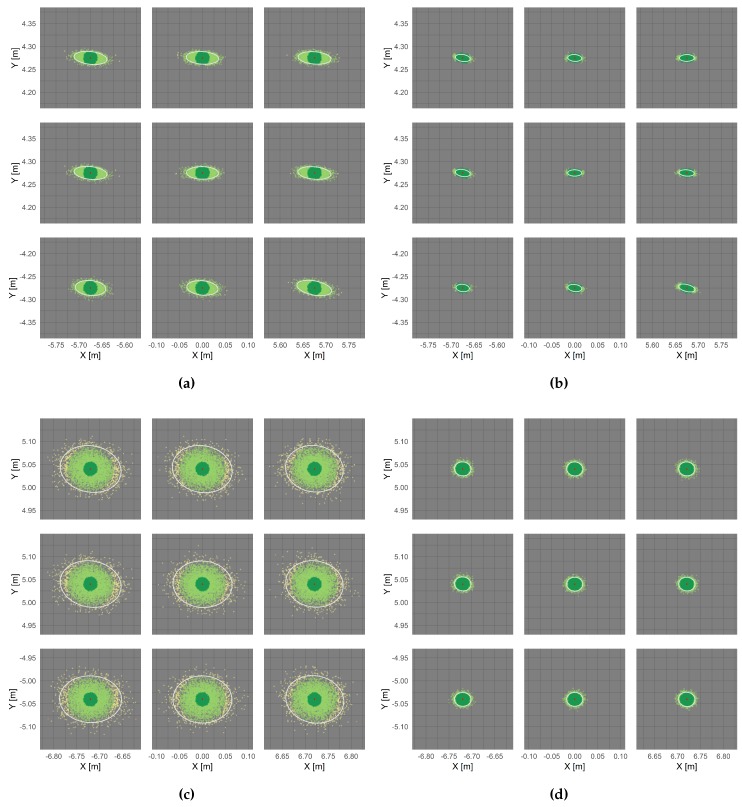

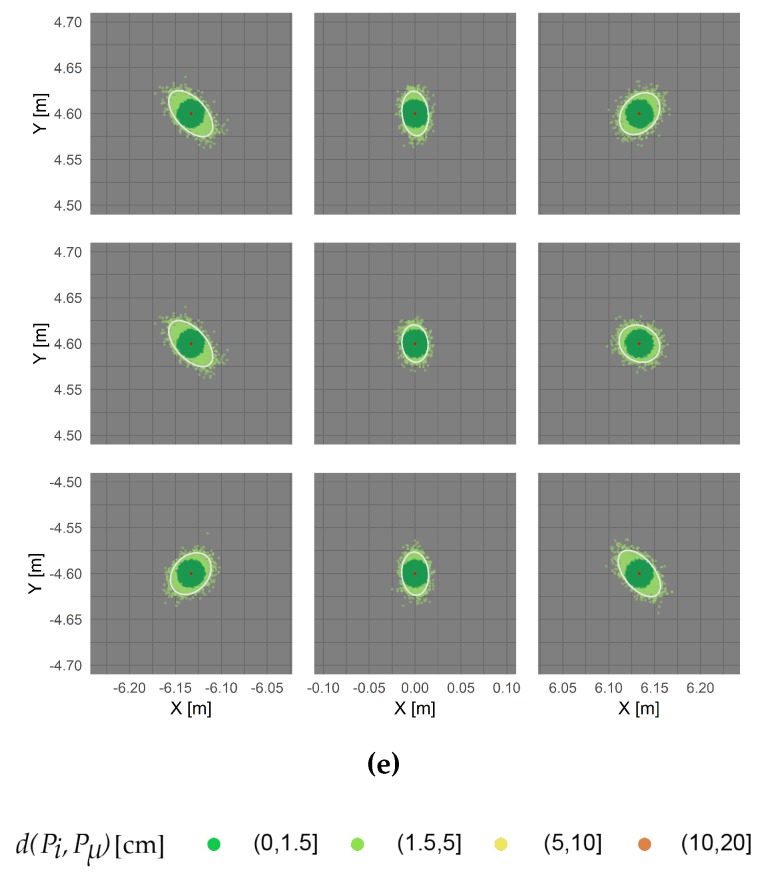
Projection of image points $p{{}^{\prime }}_{i}\left(x^{\prime },y^{\prime }\right)$ onto a virtual object plane in a distance of *Z* = 10 m with fixed EOP- and simulated IOP (visualisation of every 10th point $P_{i}\left(X,Y\right)$ ). LG Google Nexus 5, cold started (**a**) and warm started (**b**); Samsung Galaxy S8, cold started (**c**) and warm started (**d**) and Raspberry Pi v2.1 (**e**). The generated object points were colourised by means of their Euclidean distance $d(P_{i},P_{\mu })$ to the expected object point coordinates. The white ellipses are the confidence ellipses with 95% probability.

Monte-Carlo simulation was applied for each investigated camera as described in Section 3.2, i.e., 50.000 sets of modified IOP are simulated that can result from temperature change. The simulated parameters were used to project a 3 × 3 raster of image points onto a virtual object plane parallel to the camera sensor in a distance of 10 m. The results are visualised in Figure 10 where the point color refers to the Euclidean distance $d(P_{i},P_{\mu })$ between the projected object point and the expected, red-coloured object point. The Euclidean distances, which are used to determine the magnitude of errors due to temperature change, were clustered in distances <1.5 cm (dark green), 1.5–5.0 cm (light green) and 5–10 cm (yellow). Errors >10 cm (pink) appeared hardly ever.

As might be expected, the individual plots of Figure 10 reveal that cold started smartphone cameras show significantly higher errors in the point projection than warmed up cameras. Table 7 gives the percentage of point projections in relation to the visualised error clusters.

**Figure 11:**
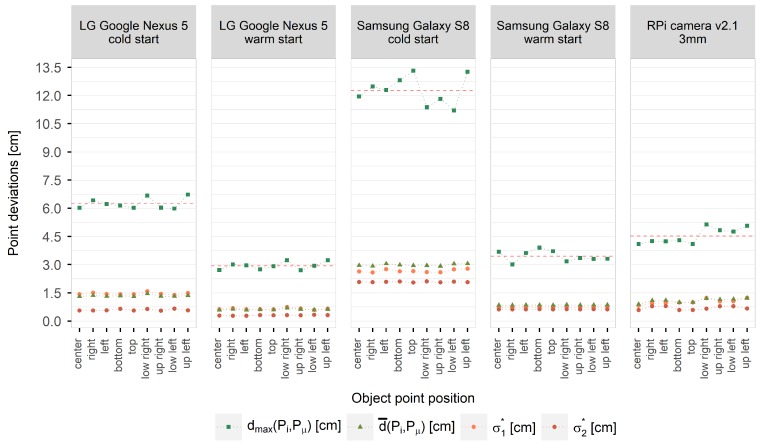
Principal standard deviations $s_{1}^{*}$ and $s_{2}^{*}$ giving the directional error in object space for each projected object point and for each investigated camera.

Considering all 50.000 iterations, the probability of temperature-related errors less than 1.5 cm amounts to 98% and 93% using the warmed up smartphone cameras of the LG Google Nexus 5 and the Samsung Galaxy S8 smartphone. Deviations of more than 5 cm are unlikely for both cameras. Similar results could be achieved for the RPi camera whose initial device temperature was similar to the device temperatures of the warm started smartphone cameras. Considering the cold-started smartphone cameras of LG Google Nexus 5 and Samsung Galaxy S8, only 67% and 20% of all projected points show deviations less than 1.5 cm. It has also been shown for the S8 camera that errors up to 10 cm are likely. Focussing on the extension and orientation of the deviations between the expected and the projected object point coordinates, which are visualised in Figure 11 by light orange $s_{1}^{*}$ and dark orange $s_{2}^{*}$ points, the errors show directionality for all investigated cameras. For the most cameras, the deviations are larger in *X*- than in *Y*-direction due to the greater scattering of the principal point in $\tilde{x}$-direction. Moreover, projected object points that originate from image points lying at the image edges and corners show higher deviations that points inside the image, which is also due to the principal distance. The changing principal distance has less impact on point projections from the image center but great impact on point projections from the image edges and corners. This becomes visible when comparing the largest deviations given by the maximum Euclidean distances $d_{max}\left(P_{i},P_{\mu }\right)$ (light green squares) in Figure 11. In relation to this, the highest deviations are shown by the cold-started smartphone cameras. Calculating the mean of the maximum deviations considering all nine projected image points (visualised in Figure 11 by a red dashed line) results in deviations of 6.2 cm and 12.9 cm for LG Google Nexus 5 and Samsung Galaxy S8, respectively.

In contrast, warm started smartphone cameras show deviations up to 2.9 cm and 3.5 cm and thus a reduced temperature-related error by half. Considering the RPi camera, the maximum deviations depend more on the image point position (lowest - image center, highest - upper right corner) with a mean of 4.5 cm. The mean of the Euclidean distances $\bar{d}\left(P_{i},P_{\mu }\right)$ between the coordinates of the projected image points in object space and the expected coordinates of the respective object points are visualised Figure 11 with dark green triangles.

To give a final magnitude of errors to be expected when the camera is exposed to changing temperature, the mean of all Euclidean distances of each projected point per investigated camera was determined (independently from the original image point position on the camera sensor). An error magnitude of 1.3 cm (cold start) and 0.6 cm (warm start) was determined for the investigated LG Google Nexus 5 camera. Furthermore, an error magnitude of about 3.0 cm (cold start) and 0.8 cm (warm start) was investigated for the applied Samsung Galaxy S8 camera. Finally, an average error of 1.1 cm was established for the used RPi camera v2.1 with a fixed focal length of 3 mm that was exposed to ambient temperature changes. Overall, the temperature-related error clearly depends on the used camera model and its construction and can be significantly reduced using warmed up devices (considering smartphone cameras). The average temperature-related measurement error that should be expected using (warmed up) cameras as measurement devices is between 1 cm and 2 cm in a camera-to-object distance of 10 m.

## 5. Conclusions

In this study, three cameras (two built in smartphones and one external RPi module) were investigated to evaluate dependencies between temperature changes resulting from self-heating or changes in the ambient temperature and the IOP. Each observed camera was installed in front of a 3D calibration test field taking serial images while the camera was exposed to temperature change. Each image was processed via spatial resection to estimate the IOP focal length, principal point, radial lens distortion and decentering lens distortion. For the smartphone cameras, which were affected by smartphone self-heating, a strong dependency between the magnitude of temperature change and the magnitude of variations of the IOP was detected. This finding was supported by visual assessment of the calibration images, which became unfocussed due to changes in the principal distances and the principal points. This effect complicated the image measurements because blurry images lead to less accurate point measurements, which was reflected in higher standard deviations of the IOP. Although the parameters reveal a linear correlation with the temperature, the rate of parameter changes is slightly different in each measurement series and for each camera, which complicates the modelling of temperature-related changes. Unfortunately, the construction of smartphone camera modules as well as their integration into smartphone bodies impedes definite statements about the physical integrity of the camera geometry. Changes in the IOP are likely due to changes of the camera module itself, such as temperature-induced tilting of the sensor plane and the projection center, or due to changes in camera’s exterior orientation due to housing deformations. Also for the RPi camera module a dependency between temperature and IOP became obvious with a strong increase of the standard deviation ${\hat{s}}_{0}$ when temperatures reached extreme values, e.g., high temperature decrease or high temperature increase. This results in both, alternating image shifts and zooming effects due to changes of the depth of the focus.

Cameras of different types show gradual warming effects that stagnate over a certain time (e.g., [14,20]), which is however not feasible in citizen science because the citizen scientists will not wait 0.5–1 h until the camera stabilises. Furthermore, smartphone temperature can change very rapidly due to varying background tasks, sensors, etc., which impedes a direct modelling of the effects of image drift. A Monte-Carlo simulation revealed that temperature-related errors between 1 cm and 2 cm at a distance of 10 m are to be expected provided that the camera is warmed up. Errors larger than 10 cm are less frequent, but should still be considered because the results of this study are rather optimistic due to the chosen reference object in form of a plane being parallel to the camera at a close distance of 10 m. Thus, it is recommended to estimate the error always in context of a specific photogrammetric application adapting the described simulation method.

In view of the above-mentioned low-cost early warning flood systems using mobile and stationary cameras, these errors should be detected and compensated in applications of long-term observations using fixed object points in river’s environment to determine the prevalent camera geometry at the time of data acquisition, e.g., using image-to-geometry registration [4,41,42]. Otherwise, image drifts can lead to false measurements of the water level, for instance considering the case that the water level increases but an image shift towards the riverbed would compensate the trend. In case of flood observation, the errors resulting from temperature-related changes in the IOP can be neglected because the reliability of water level estimation, e.g., with methods introduced by e.g., [4,43,44], decreases strongly due to large waves at the water surface that impede a unique detection of the shore line.

Further investigations will show whether, and if so, how to model temperature-related changes at the camera geometry, e.g., to enhance the reliability of low-cost water monitoring systems. Therefore, more iterations and more cameras of the same type would be advisable to detect trends in the parameters that can be assigned to the respective camera type. Furthermore, the calibration procedure can be improved using multi-image bundle adjustment using, for example, a robot controlled 3D test field that can be moved and rotated with known EOP. Thus, correlations between the IOP and EOP could be solved promising new insights at which effects are related to variations of the camera module and which effects are related to the camera module geometry. Moreover, experiments on ambient temperature changes should be repeated in a climate chamber providing full control on the temperature.

## Figures and Tables

**Table 1:** Device information and camera specifications [24,25]. Picture of Sony IMX333 Exmor RS by [26]. Abbrevations: Complementary metal-oxide-semiconductor (CMOS), Megapixel (MP).

	LG Google Nexus 5	Samsung Galaxy S8	RPi Camera v2.1
Release	October 2013	March 2017	2016
Operation system	Android 6.0.1	Android 8.0	(-)
Camera specifications			
	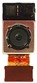	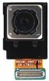	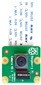
CMOS Sensor	Sony IMX179 Exmor R	Sony IMX333 Exmor RS	Sony IMX219PQ
Sensor size	4.6 mm × 3.5 mm	5.6 mm × 4.2 mm	3.7 mm × 2.8 mm
Total pixels	3288 × 2512 (8.26 MP)	-	3296 × 2512 (8.28 MP)
Active pixels	3264 × 2448 (7.99 MP)	4032 × 3024 (12.2 MP)	3280 × 2464 (8.08 MP)
Pixel size	1.40 µm × 1.40 µm	1.40 µm × 1.40 µm	1.12 µm × 1.12 µm
Focal length	3.97 mm	4.25 mm	3.0 mm

**Table 2:** Changes in the IOP of the built-in smartphone cameras from LG Google Nexus 5 and Samsung Galaxy S8 between the last and first estimated variables. $\Delta t_{cpu}$ and $\Delta t_{batt}$ are the deviations between the temperatures of the device measured at the CPU and the battery, respectively. Δk are the deviations of the estimated IOP.

	LG Google Nexus 5, Cold Started		LG Google Nexus 5, Warm Started		Samsung Galaxy S8, Cold Started		Samsung Galaxy S8, Warm Started	
	M1	M2	M1	M2	M1	M2	M1	M2
$\Delta t_{cpu}/\Delta t_{batt}$ [${}^{\circ }$C]	23.1/22.9	23.6/25.0	9.9/7.9	13.4/9.9	21.9/21.3	25.9/26.9	8.1/8.6	8.1/8.6
Δc [mm]	0.0078	0.0082	0.0028	0.0045	0.0055	0.0075	0.0014	0.0018
[Px]	5.60	5.85	2.03	3.19	3.95	5.37	1.00	1.27
$\Delta x_{0}{}^{\prime }$ [mm]	0.0205	0.0200	0.0092	0.0102	−0.0408	−0.0460	−0.0105	−0.0138
[Px]	14.62	14.32	6.56	7.28	−29.17	−32.87	−7.48	−9.87
$\Delta y_{0}{}^{\prime }$ [mm]	−0.0085	−0.0071	−0.0041	−0.0045	0.0328	0.0367	0.0077	0.0118
[Px]	−6.11	−5.11	−2.96	−3.19	23.46	26.22	5.53	8.41
$\Delta a_{1}\left[{\mathrm{mm}}^{-2}\right]$	−5.7 × 10${}^{-4}$	5.3 × 10${}^{-4}$	−5.0 × 10${}^{-4}$	3.8 × 10${}^{-4}$	−1.6 × 10${}^{-4}$	−9.8 × 10${}^{-4}$	−4.2 × 10${}^{-4}$	8.4 × 10${}^{-4}$
$\Delta a_{2}\left[{\mathrm{mm}}^{-4}\right]$	3.6 × 10${}^{-4}$	−4.1 × 10${}^{-4}$	2.4 × 10${}^{-4}$	−1.2 × 10${}^{-4}$	1.4 × 10${}^{-4}$	2.8 × 10${}^{-4}$	1.1 × 10${}^{-4}$	−2.5 × 10${}^{-4}$
$\Delta a_{3}\left[{\mathrm{mm}}^{-6}\right]$	−7.2 × 10${}^{-5}$	7.3 × 10${}^{-5}$	−3.6 × 10${}^{-5}$	−4.9 × 10${}^{-5}$	−2.0 × 10${}^{-5}$	−2.0 × 10${}^{-5}$	−9.1 × 10${}^{-6}$	2.2 × 10${}^{-5}$
$\Delta b_{1}\left[{\mathrm{mm}}^{-1}\right]$	5.9 × 10${}^{-4}$	5.8 × 10${}^{-4}$	2.5 × 10${}^{-4}$	2.7 × 10${}^{-4}$	6.9 × 10${}^{-6}$	−6.7 × 10${}^{-5}$	−2.6 × 10${}^{-5}$	4.8 × 10${}^{-5}$
$\Delta b_{2}\left[{\mathrm{mm}}^{-1}\right]$	−1.1 × 10${}^{-4}$	−1.6 × 10${}^{-4}$	−2.9 × 10${}^{-5}$	6.4 × 10${}^{-5}$	−6.0 × 10${}^{-5}$	−5.0 × 10${}^{-5}$	−2.1 × 10${}^{-5}$	−3.3 × 10${}^{-5}$
$\Delta {\hat{s}}_{0}$ [mm]	2.7 × 10${}^{-5}$	1.8 × 10${}^{-5}$	−3.6 × 10${}^{-5}$	2.7 × 10${}^{-5}$	1.7 × 10${}^{-4}$	3.0 × 10${}^{-4}$	8.0 × 10${}^{-5}$	−1.2 × 10${}^{-6}$
[Px]	0.02	0.01	−0.03	0.02	0.12	0.21	0.06	0.00

**Table 3:** Extracts from a subset of images of measurement series M1 with temperature overlay (measured at the battery). All extracts were sampled at the same image position. They reveal temperature-induced camera sensor movements and out of focus appearances due to changing temperatures.

Measurement *m*	1	25	50	75	100	125	150
LG Google Nexus 5, cold start	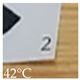	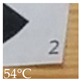	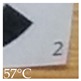	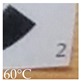	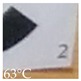	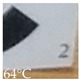	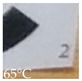
LG Google Nexus 5, warm start	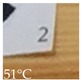	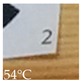	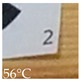	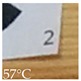	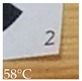	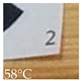	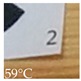
Samsung Galaxy S8, cold start	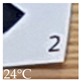	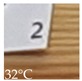	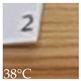	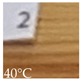	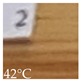	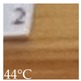	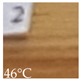
Samsung Galaxy S8, warm start	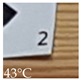	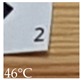	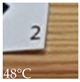	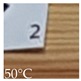	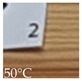	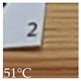	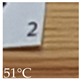

**Table 4:** F-test parameters.

Null Hypothesis	Alternative Hypothesis	Test Statistic	Critic f-Value	Rejection Criteria
$H_{0}:\sigma_{1}^{2}\leq \sigma_{2}^{2}$	$H_{1}:\sigma_{1}^{2}>\sigma_{2}^{2}$	$Q=\frac{s_{x}^{2}}{s_{y}^{2}}\left(s_{x}^{2}>s_{y}^{2}\right)$	$f_{n_{x}-1,n_{y}-1,1-\alpha }$	Q>f

**Table 5:** F-test success ratios ζ(k) to assess whether the temperature-related variance $s_{k}^{2}$ of parameter *k* is significant compared to the measurement precision ${\hat{s}}_{k}^{2}$.

	ζ(c)	$\zeta (x_{0}{}^{\prime })$	$\zeta (y_{0}{}^{\prime })$	$\zeta (a_{1})$	$\zeta (a_{2})$	$\zeta (a_{3})$	$\zeta (b_{1})$	$\zeta (b_{2})$
LG Google Nexus 5, cold started	0.94	0.90	0.88	1.00	1.00	1.00	0.86	1.00
LG Google Nexus 5, warm started	1.00	0.71	0.85	1.00	1.00	1.00	1.00	1.00
Samsung Galaxy S8, cold started	0.87	1.00	1.00	0.81	0.71	0.72	0.50	1.00
Samsung Galaxy S8, warm started	0.96	0.89	0.86	1.00	1.00	1.00	1.00	1.00
**total**	**0.94**	**0.87**	**0.90**	**0.95**	**0.93**	**0.93**	**0.84**	**1.00**

**Table 6:** Extracts from a subset of images of both measurement series M1 and M2 using the RPi camera v2.1 with a fixed focal length of 3 mm. All extracts were sampled at the same image position and superimposed with information about the prevalent temperature measured by DHT 11 sensor.

Measurement *m*	1	25	50	75	100	125	150	175	200	225	250
RPi camera v2.1, M1	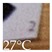	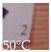	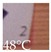	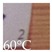	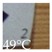	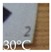	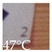	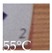	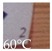	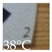	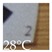
RPi camera v2.1, M2	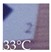	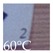	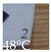	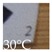	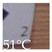	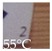	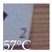	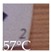	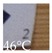	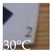	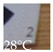

**Table 7:** Percentages of projected object points classified by their Euclidean distances to the expected object point coordinates. Clusters equal the classes used in Figure 10.

Clusters of Euclidean Distances $d(P_{i},P_{\mu })$ [cm]	(0,1.5]	(1.5,5]	(5,10]	(10,20]
LG Google Nexus 5, cold started	66.6	33.3	0.10	0.0
LG Google Nexus 5, warm started	98.0	2.0	0.0	0.0
Samsung Galaxy S8, cold started	19.5	69.7	10.8	0.0
Samsung Galaxy S8, warm started	92.6	7.40	0.0	0.0
RPi camera, 3 mm	79.4	20.6	0.0	0.0

## Abbreviations

The following abbreviations are used in this manuscript:

CMOS	complementary metal-oxide-semiconductor
CPU	central processing unit
DSLR	digital single-lens reflex
EOP	exterior orientation parameters
IOP	interior orientation parameters
MEMS	micro-electro-mechanical systems
MP	megapixel
RPi	Raspberry Pi

## Appendix A. Monte-Carlo Simulation

### Appendix A.1. General Approach of the Monte-Carlo Simulation

A vector of multivariate random numbers is used to simulate the IOP:

(A1) $$\vec{X_{k}}=\vec{\mu_{k}}+V_{k}\cdot \Lambda_{k}^{\frac{1}{2}}\cdot \vec{Z_{k}}$$

where $\vec{X_{k}}$ is the multivariate random vector of length *k* (according to the number of IOP), $\vec{\mu_{k}}$ is the vector of expected values and $V_{k}$ and $\Lambda_{k}$ are the eigenvector- and diagonal matrix that can be obtained from the variance-covariance matrix $\Sigma_{k}$ via eigen decomposition taking into account parameter correlations:

(A2) $$\Sigma_{k}=V_{k}\cdot \Lambda_{k}\cdot V_{k}^{T}$$

with:

(A3) $$\Sigma_{k}=\left[\begin{array}{cccc} \sigma_{1}^{2} & \sigma_{1}\sigma_{2}\rho_{1,2} & \cdots & \sigma_{1}\sigma_{k}\rho_{1,k}\\ \sigma_{1}\sigma_{2}\rho_{1,2} & \sigma_{2}^{2} & \cdots & \sigma_{2}\sigma_{k}\rho_{2,k}\\ \vdots & \vdots & \ddots & \vdots \\ \sigma_{1}\sigma_{k}\rho_{1,k} & \sigma_{2}\sigma_{k}\rho_{2,k} & \cdots & \sigma_{k}^{2} \end{array}\right]$$

where $\rho_{i,j}\left(i,j\in k,\rho_{i,j}\in R\right)$ are the estimated correlations between parameters *k* and $\sigma_{1},\cdots ,\sigma_{k}$ are the corresponding standard deviations. $\vec{Z_{k}}$ is the vector of *k* independent, standard normally-distributed random numbers with:

(A4) $$\vec{Z_{k}}∼N\left(0,I_{k}\right)$$

where $I_{k}$ is the identity matrix.

### Appendix A.2. Implementing the Monte-Carlo Simulation

Within the Monte-Carlo simulation $n\cdot \vec{X_{k}}$ multivariate random vectors with *n* = 50.000 iterations were calculated with random IOP using Equation (A1). Assuming a distortion-free ideal camera, the vector of expectation values $\vec{\mu_{k}}$ is defined by the principal distance $c_{m}$ given by the manufacturer specification, the image-centred principal point with $x_{0}{}^{\prime }=y_{0}{}^{\prime }=0$ and zero lens distortion:

(A5) $$\vec{\mu_{k}}={\left(c_{m},0,0,0,0,0,0,0\right)}^{T}$$

The variance-covariance matrix $\Sigma_{k}$ is based on the standard deviations $\sigma_{k}$, which are calculated from repeatedly estimated IOP of each series. Furthermore, each estimation of the IOP provides stochastic information about the correlation between the investigated IOP. $\Sigma_{k}$ uses the mean correlation coefficients $\rho_{i,j}$ that are calculated from all estimations that belong to each camera. In the case of repeated measurement periods that are made successively under similar conditions, i.e., camera, setup and the parameters to be estimated remain unchanged, the standard deviations $\sigma_{k}$ and median correlations $\rho_{i,j}$ can be combined taking their arithmetic mean (here the measurement series M1, M2 per camera).

(A6) $$\Sigma_{k}=\left[\begin{array}{cccc} \sigma_{c}^{2} & \sigma_{c}\sigma_{x_{0}{}^{\prime }}\rho_{\left(c,x_{0}{}^{\prime }\right)} & \cdots & \sigma_{c}\sigma_{b_{2}}\rho_{\left(c,b_{2}\right)}\\ \sigma_{c}\sigma_{x_{0}{}^{\prime }}\rho_{\left(c,x_{0}{}^{\prime }\right)} & \sigma_{x_{0}{}^{\prime }}^{2} & \cdots & \sigma_{x_{0}{}^{\prime }}\sigma_{b_{2}}\rho_{\left(x_{0}{}^{\prime },b_{2}\right)}\\ \vdots & \vdots & \ddots & \vdots \\ \sigma_{c}\sigma_{b_{2}}\rho_{\left(c,b_{2}\right)} & \sigma_{x_{0}{}^{\prime }}\sigma_{b_{2}}\rho_{\left(x_{0}{}^{\prime },b_{2}\right)} & \cdots & \sigma_{b_{2}}^{2} \end{array}\right]$$

with:

(A7) $$\sigma_{k}^{2}=\sum_{i=1}^{n}{\left({x_{k}}_{i}-{\bar{x}}_{k}\right)}^{2}\;\mathrm{with}\;k=c,x_{0}{}^{\prime },y_{0}{}^{\prime },a_{1},a_{2},a_{3},b_{1},b_{2}$$

(A8) $${\rho_{k}}_{i,j}=\frac{\sum_{l=1}^{n}\left({k_{i}}_{l}-{\bar{k}}_{i}\right)\left({k_{j}}_{l}-{\bar{k}}_{j}\right)}{\sqrt{\sum_{l=1}^{n}{\left({k_{i}}_{l}-{\bar{k}}_{i}\right)}^{2}\cdot \sum_{l=1}^{n}{\left({k_{j}}_{l}-{\bar{k}}_{j}\right)}^{2}}}$$

Having $n\cdot \vec{X_{k}}$ multivariate vectors of IOP, a regular grid of image points $\left(x{}^{\prime },y{}^{\prime }\right)$ is projected into object space *n*-times (see Section 3.1.1). The image points are located in the image centre, the image corners, and in the middle of the image plane edges. Considering the intersected virtual 3D object plane parallel to the sensor plane with a known depth, the EOP parameters can be simplified to:

(A9) $$R^{T}=I;\vec{X_{0}}=0$$

which results in the simplified collinearity descriptions to generate ideal image coordinates $\left(x{}^{\prime },y{}^{\prime }\right)$ in camera’s coordinate system:

(A10) $$x{}^{\prime }=-c\cdot \frac{X}{Z};\;y{}^{\prime }=-c\cdot \frac{Y}{Z}$$

However, before the image rays are generated, the image points must be distorted considering the simulated principal point, the radial lens distortion and the decentering lens distortion (see Equations (5)–(13)). Using Equations (1)–(4), the object point coordinates (X,Y,Z) can be determined from the distorted image coordinates $\left(x_{dist}{}^{\prime },y_{dist}{}^{\prime }\right)$ via projective transformation for each:

(A11) $$X=\frac{Z\cdot (x_{dist}{}^{\prime }-x_{0}{}^{\prime })}{c}$$

(A12) $$Y=\frac{Z\cdot (y_{dist}{}^{\prime }-y_{0}{}^{\prime })}{c}$$

Then, the error metric can be determined by means of the empirical variance-covariance matrices $s^{*}$ that are determined for each point of the image grid transferred into object space:

(A13) $$\Sigma^{*}=\left[\begin{array}{cc} s_{X}^{*2} & s_{XY}^{*}\\ s_{XY}^{*} & s_{Y}^{*2} \end{array}\right]$$

with:

(A14) $$s_{X}^{*2}=\frac{1}{n}\cdot \sum_{i=1}^{n}{\left(X_{i}-\mu_{X}\right)}^{2}$$

(A15) $$s_{Y}^{*2}=\frac{1}{n}\cdot \sum_{i=1}^{n}{\left(Y_{i}-\mu_{Y}\right)}^{2}$$

(A16) $$s_{XY}^{*}=\frac{1}{n}\cdot \sum_{i=1}^{n}\left(X_{i}-\mu_{X}\right)\cdot \left(Y_{i}-\mu_{Y}\right)$$

where $s_{X}^{*2},s_{Y}^{*2}$ are the variances of the distorted object point coordinates $P_{i}\left(X_{i},Y_{i}\right)$ to the ideal, error-free object point coordinates $P_{\mu }\left(\mu_{X},\mu_{Y}\right)$ and $s_{XY}^{*}$ are the corresponding covariances. Performing eigenvalue decomposition of $\Sigma^{*}$ enables the determination of the principal standard deviations $s_{1}^{*},s_{2}^{*}$, which are used as quality measures:

(A17) $$\Sigma^{*}=V^{*}\cdot \Lambda^{*}\cdot V^{*T}=V^{*}\cdot \left[\begin{array}{cc} s_{1}^{*2} & 0\\ 0 & s_{2}^{*2} \end{array}\right]\cdot V^{*T}$$

where $\Lambda^{*}$ represents the diagonal eigenvalue matrix and $V^{*}$ represents the eigenvector matrix. Also, the Euclidean distances $d(P_{i},P_{\mu })$ are calculated between the distorted- and the error-free object coordinates with

(A18) $$d\left(P_{i},P_{\mu }\right)=∥P_{i}-P_{\mu }∥$$

to quantify the accuracy using the mean and maximum distances $\bar{d}\left(P_{i},P_{\mu }\right)$ and $d_{max}\left(P_{i},P_{\mu }\right)$, respectively.
